# Changes in Antioxidant Defence System in Durum Wheat under Hyperosmotic Stress: A Concise Overview

**DOI:** 10.3390/plants11010098

**Published:** 2021-12-29

**Authors:** Maura Nicoletta Laus, Michele Andrea De Santis, Zina Flagella, Mario Soccio

**Affiliations:** Department of Agriculture, Food, Natural Resources and Engineering (DAFNE), University of Foggia, Via Napoli, 25, 71122 Foggia, Italy; maura.laus@unifg.it (M.N.L.); michele.desantis@unifg.it (M.A.D.S.)

**Keywords:** durum wheat, drought, water stress, salt stress, hyperosmotic stress, oxidative stress, ascorbate-glutathione cycle, superoxide dismutase, catalase, peroxidase

## Abstract

Durum wheat is one of the most commonly cultivated species in the world and represents a key commodity for many areas worldwide, as its grain is used for production of many foods, such as pasta, bread, couscous, and bourghul. Durum wheat grain has a relevant role in the human diet, providing carbohydrates, proteins, lipids, fibres, vitamins, and minerals, as well as highly valued bioactive compounds contributing to a healthy diet. Durum wheat is largely cultivated in the Mediterranean basin, where it is mainly grown under rain-fed conditions, thus currently undergoing drought stress, as well as soil salinity, which can hamper yield potential and influence the qualitative characteristics of grain. When plants suffer drought and/or salinity stress, a condition known as hyperosmotic stress is established at cellular level. This leads to the accumulation of ROS thus generating in turn an oxidative stress condition, which can ultimately result in the impairment of cellular integrity and functionality. To counteract oxidative damage due to excessive ROS production under stress, plants have evolved a complex array of both enzymatic and non-enzymatic antioxidant mechanisms, working jointly and synergically for maintenance of ROS homeostasis. Enhancement of antioxidant defence system has been demonstrated as an adaptive mechanism associated to an increased tolerance to hyperosmotic stress. In the light of these considerations, this review provides a concise overview on recent advancements regarding the role of the ascorbate-glutathione cycle and the main antioxidant enzymes (superoxide dismutase, catalase, and peroxidases) in durum wheat response to drought and salt stresses that are expected to become more and more frequent due to the ongoing climate changes.

## 1. Introduction

### 1.1. Geographic Diffusion and Nutritional Relevance of Durum Wheat

Durum wheat (*Triticum turgidum* L. subsp. *durum* Desf.) is the 10th most important and commonly cultivated species in the world, with a planting area of 17 million hectares globally and a yearly production average of 38 million tons in 2019 [1]. The EU is the primary producer in the world and Italy is the leading European country in durum wheat production, followed by France and Turkey (International Grains Council, https://www.igc.int/en/default.aspx, accessed on 10 May 2021).

Durum wheat is cultivated in the northern United States and Canada, and within the desert areas of the south-west United States, northern Mexico, and sub-Saharan Africa [2], but most of its production process takes place in the countries surrounding the Mediterranean basin. Other much smaller areas where durum wheat is cultivated include Russia and Kazakhstan, Australia, India, and Argentina.

This species is a key commodity for many areas worldwide, as its grain is used for production of many foods, having a large diversification across the producing countries. Pasta is the most popular durum-based product worldwide, couscous and bulgur are common durum-based food in North Africa and in the Mid-East, respectively, and durum wheat breads are traditionally important in Southern Italy, Spain, Turkey, and Mid-East Mediterranean regions.

Durum wheat grain is an important food source with a relevant role in the human diet, providing carbohydrates (70.2%), proteins (12.2%), lipids (1.9%), fibre (1.6%) and minerals (1.6%) [3]. Additionally, wholegrain is also a source of bioactive components contributing to a healthy diet having high antioxidant levels, such as carotenoid pigments and polyphenols, together with higher vitamin, sodium, potassium, calcium, and magnesium contents, compared with other cereals [3].

It should be outlined that, in the Mediterranean environments, durum wheat is mainly grown under rain-fed conditions, thus often encountering drought stress that hamper yield potential and influence the grain qualitative characteristics [4,5]. In the coastal areas of the Mediterranean basin, durum wheat plants currently undergo also increasing salinity due to salt accumulation in the soil as a consequence of both the seawater intrusion into fresh water aquifers and the use of salty water for irrigation [6,7,8].

### 1.2. Drought and Salinity: Impact on Plant Physiology in Relation to Antioxidant Defence System

#### 1.2.1. Drought and Salinity in the Global Climate Change Scenario

How much climate change will affect the production and the quality of crops is an overarching question. The past decade has been identified as the warmest on record, and the Inter-governmental Panel on Climate Change (IPCC) predicts that by 2050, global average temperature may rise as much as 2 °C over pre-industry levels (and nearly 5 °C by 2100) ([9] and refs therein). Moreover, the global Mediterranean-like climates are hot-spots of climate change, where temperature is warming faster than in other world regions [10].

Due to changing climate, environmental constraints, especially drought, high soil salinity, temperature fluctuations, and ultraviolet radiation, are more and more frequent and have become the most critical factors that severely reduce crop productivity [11,12]. 

This poses a serious challenge for plant growth and productivity of agricultural crops. Extremely high stress levels or adverse environmental factors extended for prolonged periods negatively affect plant growth and development, thus leading to reduced crop yield and harvest quality, and in extreme conditions, to plant death [13,14]. Abiotic stresses are jeopardising agricultural food production on global scale, and so plants’ responses to stressful growth conditions are topics that increasingly attract researchers’ attention worldwide [15].

Drought stress is the abiotic stress causing major setbacks to agricultural productivity every year. Drought conditions may induce crop failures, severely affecting food production all over the world. Agricultural drought is associated with decreased soil water levels due to decline in rainfall and increased frequency of dry spells [11]. The complexity of drought stress is aggravated by many phenomena, which also influence its severity. These include water deficit, low atmospheric moisture content, intense radiation, heat shock and high salt concentration, all of which interact with the soil type [16,17]. Drought is often accompanied by other detrimental effects, like salinity and heat. The combined occurrence of different types of abiotic stress may have an additive effect, thus causing much greater damage. Such concurrences are more deleterious than drought occurring alone and may lead to great agricultural damage ([14] and refs therein). It should be also considered that global climate change is expected to accelerate in the future, thus inducing prevalent drought stress conditions over vast areas at a global scale.

Salinity, which leads to permanent modifications of the physical–chemical properties of the soil [18], is another detrimental abiotic stress, which poses a major threat to sustainable crop production in many parts of the world by limiting plant growth and productivity of agricultural crops ([13] and refs. therein). It is a frequent stress condition, considering that more than 6% of the world’s arable land is affected by salinity or sodicity, and a significant proportion of agricultural soil has become saline due to land clearance and irrigation [19,20]. This problem is becoming more serious due to the effect of climate change that is expected to further exacerbate seawater intrusion due to sea-level rise [21]. Consequently, salinity has become an increasingly widespread problem, and it is believed that, by 2050, salinization will affect more than 50% of all arable lands [22]. It should be also considered that in regions of high salinity, occurrence of drought is also frequent, because as the soluble solute levels increase, water uptake becomes limited, thus initiating drought conditions [11].

#### 1.2.2. Physiological and Biochemical Responses to Hyperosmotic Stress: Focus on the Role of Antioxidant Defence System in Stress Tolerance

Salinity and drought exhibit a great degree of similarity in their physiological, bio-chemical, molecular, and genetic effects [11], depending not only on the intensity and duration of the stress, but also on the developmental stage of the plant and on the genetically determined stress tolerance [23,24].

A decrease in soil water potential due to scarce water availability and/or to increased soil salt concentration induces a decreased ability of a plant to take up water, thus establishing a condition known as hyperosmotic stress or simply osmotic stress [25]. It leads to a decreased cell turgor, which induces several physiological and morphological modifications like reduced transpiration and photosynthesis rate, osmotic adjustments, repressed root and shoot growth, modified stress signalling pathways, and senescence [11]. In addition, an increase in soil salinity level leads to an increase in endogenous sodium (Na^+^) and chloride (Cl^−^) contents, which can create life-threatening conditions for plants (ion stress and toxicity) and ion/nutrient imbalance, due to unfavourable effect of Na^+^ on K^+^ nutrition and on cytosolic enzymes activities [13].

An in-depth discussion of hyperosmotic stress impacts in plants is reported in [11,13,26,27,28,29,30].

Moreover, salinity- and drought-induced osmotic effects alter general metabolic processes and enzymatic activities leading to an enhancement in generation of reactive oxygen species (ROS). The major ROS include hydrogen peroxide (H_2_O_2_), superoxide anion (O_2_·^−^), hydroxyl radical (·OH), and singlet oxygen (^1^O_2_), each showing a characteristic half-life and oxidizing potential [13,31,32]. The generation of toxic ROS as a by-product of metabolic processes occurs in various cellular sites, such as mitochondria, chloroplast, peroxisome, and apoplast. It has been reported that at low concentration ROS have important roles in plant cells, such as control and regulation of cell growth, cell cycle, programmed cell death, hormone signaling, plant responses to biotic and abiotic stress, and development of tissues [33]. Anyway, when ROS production exceeds plant ability to scavenge them, a condition known as oxidative stress is established inside the cell, characterized by a rapid leakage of excess ROS into other parts of the plant [14]. Elevated ROS levels are harmful to plants, since these toxic molecules can cause oxidative damage to membrane lipids by lipid peroxidation, to nucleic acids, and to enzyme by protein oxidation, thus leading to the impairment of cellular integrity and functionality and promoting programmed cell death [31].

To combat oxidative damage due to excessive ROS production, plants have evolved a complex array of both enzymatic and non-enzymatic detoxification mechanisms, working jointly to maintain redox homeostasis by minimizing, buffering, and efficiently scavenging ROS [34,35]. The foremost prominent non-enzymatic antioxidants include water-soluble molecules, such as ascorbate (AsA), reduced glutathione (GSH) and phenols, as well as liposoluble compounds such as tocols and carotenoids [13,31]. These molecules can react directly with ROS and scavenge them, thus playing a vital role in plant protection from ROS-induced oxidative stress and in enhancing the plant tolerance against stress. Nevertheless, the efficiency of ROS removal is much higher in reactions mediated by enzymatic systems ([36] and refs therein). In plants, the foremost relevant antioxidant enzymes include superoxide dismutase (SOD), catalase (CAT), ascorbate peroxidase (APX), dehydroascorbate reductase (DHAR), monodehydroascorbate reductase (MDHAR), and glutathione reductase (GR).

SOD (EC 1.15.1.1) is a metallo-enzyme acting as the first level of protection against ROS, being involved in scavenging of O_2_·^−^ by dismutation to H_2_O_2_ and O_2_ ([37] and refs therein). Counting on metal cofactor present within the active centre, three main SOD types are described in plants: Cu/Zn-SOD (present in cytosol, chloroplast stroma, peroxisomes, and apoplast), Mn-SOD (found in mitochondria, peroxisomes, apoplast, and cell wall), and Fe-SOD (restricted to chloroplast stroma of certain plant species) [36,38]. SOD works in close synchrony with other antioxidant enzymes, such as CAT, peroxidases (POX), and APX, able to detoxify the resulting H_2_O_2_ thus preventing formation of other harmful ROS such as OH. CAT (EC 1.11.1.6) is a haem-containing enzyme catalyzing the rapid H_2_O_2_ decomposition to H_2_O and O_2_. It is present in large quantities in peroxisomes, where it scavenges H_2_O_2_ generated during photorespiration and β-oxidation of fatty acids, but it has been also found in mitochondria and cytosol, but not in chloroplasts [27,36]. Hydrogen peroxide is more efficiently scavenged by APX, which is a widely distributed enzyme and has a better affinity for H_2_O_2_ than CAT. APX is the most important antioxidant enzyme of the AsA-GSH cycle [37], operating in H_2_O_2_ removal mainly in chloroplasts, where CAT is absent, as well as in other cellular compartments including cytosol, mitochondria, and peroxisomes [36]. In this pathway, APX uses AsA as specific electron donor to reduce H_2_O_2_ to water; then, AsA is regenerated from monodehydroascorbate (MDHA) and dehydroascorbate (DHA) via MDHAR and DHAR using NAD(P)H and GSH, respectively; finally, GSSG is reduced via GR, using NAD(P)H as reducing power [36]. Hydrogen peroxide is also eliminated by peroxidases (POX), glycoproteins catalyzing reduction of H_2_O_2_ to H_2_O using electrons from various reductants, including phenolic and non-phenolic substrates [39,40,41]. Interestingly, changes in expression/activity of antioxidant enzymes in response to oxidative stress induced by plant exposure to abiotic stress has been reported in various species [37,40]. Overexpression of antioxidant enzymes may be considered a potential stress biomarker [40]; enhanced activity of antioxidant enzymes represent an adaptive mechanism associated to increased plant tolerance to environmental stress [36,42].

In the light of the economic and nutritional relevance of durum wheat and of the pivotal role of antioxidant defence system in plant adaptation to hyperosmotic stress, this review provides concise overview of recent findings related to change in antioxidant network in durum wheat plants under drought and salt stresses.

## 2. Effect of Hyperosmotic Stress on Antioxidant Defence System in Durum Wheat

Studies reported in this section are critically discussed in relation to the comparison among durum wheat genotypes with different stress sensitivity and/or of durum wheat with other cereal species, as well as by taking into account the severity and duration of stress, the type of tissue/organ, and the stage of development.

Details of experimental conditions applied in these studies, as well as the main interesting results relative to antioxidant response, are summarized in Table 1 (drought) and Table 2 (salinity).

### 2.1. Drought Stress

Few literature studies examined antioxidant responses of durum wheat to drought stress imposed under controlled conditions (growth chambers, greenhouses). These studies focused on comparison among different durum wheat genotypes at early vegetative stages (at young seedling stage) (Table 1).

**Table 1:** Changes in the antioxidant defence system in durum wheat under drought stress. Effects were estimated from graphs, figures and tables if not directly given in the text or supplements.

Plant Species	Growth Conditions	Stress Treatment/s	Tissue	Changes in Antioxidant Components	Ref.
**50 durum wheat genotypes** 18 Iranian landraces, 3 local Kermanshah, Iran and 29 breeding lines	Growth chamber	PEG-6000 (imposed at the three-leaf stage) for 14 days	Leaf obtained 14 days after PEG treatment	**SOD** activity: on average +92% **CAT** activity: on average +11% **APX** activity: on average +15% **POX** activity: on average +177%	[43]
**1 durum wheat genotype** Maali **3 T3 transgenic durum wheat lines** overexpressing TdPIP2	Greenhouse	40% of field capacity imposed after 2 weeks from sowing until the end of plant cycle	Leaf	**CAT** activity: about 1.6- and up to 2.2-fold increase in wild type transgenic lines, respectively **SOD** activity: about 1.7- and up to 3.2-fold increase in wild type and transgenic lines, respectively	[44]
**8 durum wheat near-isogenic lines** derived from 4 different recombinant inbred lines of a cross between Kofa and Svevo	Field	Rain-fed conditions and two different sowing densities (480 seeds/m^2^ and 320 seeds/m^2^)	Leaf at flowering	**APX** activity: on average 70% decrease **POX** activity: on average 50% increase	[45]
**2 durum wheat genotypes** Barakatli-95 (drought-tolerant) Garagylchyg-2 (drought-sensitive) **2 bread wheat genotypes** Giymatli-2/17 (drought-sensitive) Azamatli-95 (less drought-sensitive)	Field	Rain-fed conditions imposed by withholding irrigation	Leaf at seven different stages of ontogenesis	**APX** activity: on average about 1.4- fold increase and 30% decrease in Barakatli-95 and Garagylchyg-2, respectively, with the maximal activity under stress at the end of flowering and of earing, respectively **SOD** activity: on average no significant change and about 60% decrease in Barakatli-95 and Garagylchyg-2, respectively **CAT** activity: on average 1.75- and 1.2-fold increase in Barakatli-95 and Garagylchyg-2, respectively, with the maximal activity at the milk ripeness stage **GR** activity: increase in Barakatli-95 at all stages of ontogenesis, with the maximal activity under normal water supply at flowering For bread wheat genotypes see literature	[46]
**2 durum wheat genotypes** Barakatli-95 (drought-tolerant) Garagylchyg- 2 (drought-sensitive)	Field	Rain-fed conditions imposed by ceasing watering	Leaf at seven different stages of ontogenesis	**SOD** activity: on average no significant change and about 60% decrease in Barakatli-95 and Garagylchyg-2, respectively **Changes in SOD isoenzyme composition under stress**	[47]
**2 durum wheat genotypes** Barakatli 95 (drought-tolerant) Garagylchyg-2 (drought-sensitive)	Field	Rain-fed conditions (imposed by ceasing watering from April to June)	Leaf and root at three different stages of ontogenesis	**APX** activity: on average no significant change in leaves Barakatli-95 and about 1.2-fold increase in leaves and roots of Garagylchyg-2, with the maximal activity at flowering **Guaiacol-type POX** activity: on average about 7-fold increase in leaves and roots of Barakatli-95 and 1.5-fold increase in leaves and about 50% decrease in roots of Garagylchyg-2, with the maximal activity in leaves and roots at wax ripeness and flowering, respectively **Benzidine-type POX** activity: on average 2.3- and 1.8-fold increase in leaves and roots of Barakatli-95, respectively, and 1.6- and 1.4-fold increase in leaves and roots of Garagylchyg-2, respectively, with the maximal activity at wax ripeness **CAT** activity: on average 1.4- and 1.2-fold increase in leaves of Barakatli-95 and Garagylchyg-2, respectively, and no significant change and about 20% decrease in roots of Barakatli-95 and Garagylchyg-2, respectively	[48]
**2 durum wheat genotypes** Barakatli-95 (drought-tolerant) Garagylchyg-2 (drought-sensitive)	Field	Rain-fed conditions (imposed by ceasing watering from April to June)	Leaf and roots at three different stages of ontogenesis	**AsA** content: similar decrease in all stages of ontogenesis ranging from 20% to 25% in leaves and roots of both Barakatli-95 and Garagylchyg-2	[49]
**1 durum wheat genotype** A 9-30-1 **1 *Triticum dicoccum* genotype** HW 24 **2 bread wheat genotypes** C 306 (drought-resistant) and Hira (drought-sensitive)	Natural conditions in earthen pots	Drought stress imposed by withholding water supply for 7 days during 3 different phases, at 50% anthesis and 10 and 20 days after anthesis (DAA)	Leaf at 7, 17 and 27 DAA	**SOD** activity: up to about 30% increase at 27 DAA in durum wheat **CAT** activity: slight increase in durum wheat **POX** activity: slight increase in durum wheat For the other cereal species, see literature	[50]
**3 durum wheat genotypes** Kızıltan-91, Kunduru 414-44 and Ç.1252 **3 bread wheat genotypes** Bezostaya-1, Seri-82 and Kıraç-66	Growth chamber	Drought stress (imposed on 6-day-old seedlings), in combination with low (5/−5 °C, day/night) or high (40/30 °C) temperatures conditions, for 6 days	Leaf from 12-day-old seedlings	**CAT** activity: no significant change in Kızıltan-91, and about 30% increase and 40% decrease in Kunduru 414-44 and Ç.1252, respectively, under drought stress and normal temperature **GR** activity: no significant change in Ç.1252 and about 2- and 1.4-fold increase in Kızıltan-91 and Kunduru 414-44, respectively, under drought stress and normal temperature **AsA+DHA** content: about 30% increase in Kızıltan-91 and about 25% decrease in both Kunduru 414-44 and Ç.1252, under drought stress and normal temperature For combined effects of drought and low or high temperatures, see literature	[51]

About this point, Pour-Aboughadareh et al. [43], who examined 50 durum wheat genotypes, including ICARDA breeding lines, Iranian landraces and cultivars, found significant average increase of POX (evaluated as guaiacol-dependent activity), SOD, APX, and CAT activities of about +170%, +90%, +15%, and +10%, respectively, in leaf tissues obtained from 14-day polyethylene glycol (PEG-6000)-stressed plants. All investigated genotypes also displayed a decreasing trend of biomass (e.g., root and shoot dry weights) and physiological traits (e.g., photosynthetic pigment content and leaf Relative Water Content, RWC) under water stress [43]. Interestingly, the same authors reported a significant correlation between CAT, SOD, and APX activities, as well as root and shoot dry weights, with the stress tolerance index, thus suggesting that biomass and enzymatic antioxidant parameters could be considered as reliable markers for screening wheat genotypes for tolerance to PEG-induced water stress during early growth stage [43]. Likewise, in the study of Ayadi et al. [44], CAT and SOD activities were found to increase under 2-week drought stress (40% of field capacity in greenhouse experiment) in leaves of both wild-type plants and three homozygous T3 transgenic durum wheat lines overexpressing the wheat plasma membrane aquaporin *TdPIP2*. Interestingly, this increase in CAT and SOD activities was much higher in transgenic lines (up to about 2.2- and 3.2-fold compared to the control, respectively) than in wild-type plants, and it was associated with lower malondialdehyde (MDA) and H_2_O_2_ contents, thus suggesting a higher capacity of transgenic plants to scavenge ROS than the wild-type ones. The transgenic wheat lines also exhibited higher adaptability to drought than the wild-type plants, as indicated by improved germination rates and biomass production, as well as the capability to reach maturity, to flower, and to set grains under drought [44].

Most of the studies regarding the impact of drought stress on wheat antioxidant system were performed in field by comparing results obtained under control well-watered and water deficit (imposed by withholding water supply) treatments. Due to the multiplicity of factors influencing field experiments, these studies provided, in some cases, contrasting results with each other and compared to controlled-environment studies. In this regard, Bányai et al. [45] evaluated the antioxidant response under rain-fed and well-watered conditions in the flag leaf at the flowering stage of eight near-isogenic lines of spring durum wheat, selected for yield QTLs. Authors reported a significant increase (on average) of POX activity in the non-irrigated treatment compared to irrigated plots, while a decrease of APX activity was observed, resulting of a similar order of magnitude for all the isogenic lines (averaging about 70%) [45]. Interestingly, APX activity was in significant positive correlation with the number of spikelets on the main spike (*r* = 0.803***), and POX activity with the number of sterile basal spikelets (*r* = 0.847***) [45].

An in-depth investigation was performed by Huseynova and co-workers [46,47,48,49], who compared antioxidant behaviour under rain-fed and well-watered field conditions of wheat genotypes differing in drought tolerance, also considering different plant organs (leaves and roots), obtained at different both vegetative and reproductive growth stages. As for genotypic comparison, a generally more efficient response of antioxidant system was observed under water deficit in the drought-tolerant durum wheat variety Barakatli-95 than in the drought-sensitive Garagylchyg-2. Indeed, the authors reported a more substantial increase of CAT activity under drought during ontogenesis in the leaves of the drought-tolerant genotype as compared to the sensitive one [46,48]. Water deficit also increased APX and GR activities in the leaves of the tolerant variety, while no significant changes of GR activity, as well as a decrease of APX activity, were observed under drought in the susceptible genotype [46]. As for the impact of developmental stage, relevant stress-induced changes of antioxidant performance were generally observed in the reproductive phases of flowering, milk ripeness and wax ripeness, at which plants are more exposed and sensitive to water deficiency. About this, the authors detected maximal activities of APX, CAT, and SOD in the drought-tolerant variety at the end of flowering, milky ripeness and wax ripeness, respectively [46,47,48]. On the other hand, the drought-sensitive variety displayed the lowest SOD and GR activities at waxy ripeness, as well the minimal APX activity at milky ripeness [46,48]. As for the antioxidant behaviour of different plant tissues under water deficit, the authors found generally different or minor variations of antioxidant response in roots compared to leaves. In this regard, under an adequate water supply, a lower constitutive level of CAT and APX activities was registered in roots compared to leaves in both varieties. In addition, less marked changes in enzymatic activities (APX) and/or a different pattern of variations (CAT, POX) during ontogenesis were observed in water-stressed roots as compared to leaves [48]. A generally lower ascorbate content was found in roots compared to leaves of both genotypes; moreover, in the waxy ripeness phase water stress significantly reduced ascorbate content in leaves of both genotypes, while no significant effects were found in roots [49]. It should be outlined that the tolerant Barakatli-95 cv. showed a lower decrease in leaf RWC compared to the sensitive Garagylchyg-2 [46]. Barakatli-95 also exhibited the capacity to preserve the photosynthetic apparatus, as indicated by lower decrease in pigment content under drought than Garagylchyg-2, as well as the ability to maintain a high photosynthetic activity under stress due to a much less marked reduction of PS II activity at all stages of ontogenesis [46,48]. Moreover, the drought-resistant variety showed the capability to quickly reorganize the photosynthetic apparatus under drought conditions for adaptation to external stress [49]. The analysis of isoenzyme profiles also revealed a heterogeneity of individual forms of enzymes that authors suggested might ensure rapid plant adaptation to soil water shortage [48]. In particular, nine isoenzymes of SOD showing different subcellular localizations were detected in leaves of both control and stressed plants: three Mn-SOD (mitochondrial fraction), preferentially enhanced by drought stress, one Fe-SOD (chloroplast fraction), and five Cu/Zn-SOD (all subcellular fractions) [47]. Moreover, one isoform of CAT and seven isoforms of APX (four at the flowering and three at the wax ripeness) were detected in leaves, as well as four isoforms of APX in roots, under both control and soil drought conditions [48]. Interestingly, one CAT isoform was found in the roots under normal water supply and three isoforms were observed under drought stress condition [48].

Another study examined antioxidant response to 7 days of drought stress in different developmental stages (50% anthesis and 10 and 20 days after anthesis) by comparing durum wheat (cv. A 9-30-1) with other cereal species, such as the tetraploid *Triticum dicoccum* (cv. HW 24) and two hexaploid *Triticum aestivum* (cvs. C 306 and Hira, drought-resistant and drought-susceptible, respectively) genotypes [50]. Interestingly, water stress induced a significant increase of the activity of SOD, CAT, and POX in all species under study [50]. Nevertheless, both tetraploid genotypes showed higher SOD activity under irrigated conditions as compared to hexaploid cultivars, as well a higher increase under drought stress with respect to irrigated control [50]; however, CAT and POX activities resulted higher in the hexaploid genotypes than in the tetraploid ones [50]. Compared to hexaploids, both tetraploid genotypes also exhibited a lower reduction of total dry matter and retained higher leaf RWC under stress at all investigated stages, associated to lower H_2_O_2_ and MDA contents, and a less marked reduction of membrane stability index [50]. In the study of Keleş and Öncel [51], the antioxidant response of three different durum wheat genotypes was compared to that of three bread wheat cvs. in relation to the combined application of drought and temperature stress. Under low (5/−5 °C, day/night) temperature–drought combination, lower CAT activities were measured in durum than bread wheat genotypes; CAT activity reached the lowest values under high (40/30 °C) temperature–water stress conditions in all genotypes. On the contrary, GR activity was activated by drought stress under normal (24/16 °C) temperature, and it significantly increased under high temperature–water stress combination in all genotypes. Low temperature–drought stress combination had no effect on total ascorbic acid content, and very few changes were observed under high temperatures [51]. A relationship between the severity of water stress and elongation of seedlings was also observed [51].

Overall, the results of the studies reported in this section clearly highlighted a univocal and concordant enhanced antioxidant response in durum wheat plants exposed at young seedling stage to water deficit under controlled experimental conditions, resulting greatly genetically determined and generally exhibiting a reverse behaviour compared with biomass and physiological traits. On the other hand, a more complex response of antioxidant defence system under soil drought is pointed out by rain-fed field experiments, which highlight different dynamics for the different types of antioxidant enzymes, largely dependent on wheat genotype, but also greatly influenced by plant organs and developmental stage of stressed plants. Distinct qualitative and quantitative variations in antioxidant enzymes involved in H_2_O_2_ neutralization were also described in durum wheat under water stress, with a leading role attributed by Huseynova et al. [48] to CAT as compared to POX in the tolerant genotype. Durum wheat plants also showed a differential pattern of changes in antioxidant enzymes performing the same catalytic function, with the appearance of additional APX isoforms in the ripeness phase and the formation of new CAT isoforms in the stressed roots [48]. A differential regulation under water deficit of activities of antioxidant enzyme isoforms with different subcellular localization was also reported, with the greatest contribution to stress-induced increase of total SOD activity given by mitochondrial Mn-SOD enzyme [47]. Moreover, relevant differences appear in durum wheat response to water stress in the case of environmental stress combinations, with a loss of CAT activity and enhanced activity of the Halliwell/Asada pathway enzymes (GR and APX) under drought and high temperatures interaction [51].

### 2.2. Salinity Stress

Most of the studies referred to salt stress investigated the behaviour of antioxidant defence system under controlled conditions (growth chamber/greenhouse/phytotron) in durum wheat plants at the vegetative stages, by comparing cultivars with different salt sensitivity and/or other cereal species, as well as by taking into account the type of tissue/organ, the severity and duration of stress (Table 2). In this regard, Feki et al. [52] reported a more marked increase of SOD, CAT, APX and POX activities following 3-day treatment with increasing NaCl concentrations in the shoot of the salt-tolerant Tunisian durum wheat cv. Om Rabia3 than in the salt-sensitive Mahmoudi. Moreover, in the same study, a lower transcript accumulation of *CAT*, *Mn*-*SOD* and *APX* genes was measured in this salt-sensitive genotype as compared to the tolerant one, as well as a higher increase in MDA, H_2_O_2_ and O_2_·^−^ content and a significant decrease in AsA, associated with a higher decrease in dry biomass in both roots and shoots. Nevertheless, it should be outlined that a less efficient response to salt stress was observed in Om Rabia3 genotype when it was compared to the diploid einkorn wheat cv. Turkey [52]. A differential response to salt stress treatment of glutathione pool was observed in two different tissues (leaves and roots) of the Tunisian cvs. Karim (tolerant) and Azizi (sensitive) [53]. In particular, a more marked decrease of GSH content was found in both leaves and roots of the tolerant genotype (about 80% and 65%, respectively) than the sensitive one (about 35% and 45%, respectively), which could explain the important role of this tripeptide in maintaining the activity levels of GSH-dependent enzymes. Moreover, under salinity stress conditions cv. Karim showed higher levels of biomass production and chlorophyll content when compared with Azizi. On the contrary, no significant change in GSSG content was observed in Karim, while GSSG decreased (50%) in the roots and remained unchanged in the leaves of Azizi [53]. A different capability to react against salt stress was also observed between the drought-tolerant cv. Ofanto and the more sensitive cv. Adamello [54]. In particular, despite a higher constitutional content of ascorbate pool in Adamello, under salt stress, Ofanto resulted able to induce ascorbate synthesis and showed higher turnover rates. On the other hand, salt stress imposition induced an increase in GSH content in the roots of both genotypes, likely for an increased requirement of antioxidants in the organs that firstly suffer stress, but only roots of Ofanto showed GSH oxidation. As for the activity of the antioxidant enzymes (APX, MDHAR, DHAR, GR), the two different tested tissues showed an opposite pattern with a general increasing trend in the shoots, as well as a decreasing trend in the roots [54].

It should be underlined that the salt stress response of antioxidant system was investigated in Ofanto by considering not only the type of tissue/organ (roots and shoots), but also the duration of salt stress treatment. With this regard, D’Amico et al. [55] reported a significant increase of APX activity at 21 days of salt treatment compared to 14 days in both roots and shoots, while GR activity highly increased in roots (about 8- and 4-fold at 14 and 21 days, respectively), but decreased in shoots (about 35% and 50% at 14 and 21 days, respectively). Moreover, as for the antioxidant molecules, the ascorbate pool exerted its positive influence especially in shoots, where the ratio AsA/DHA showed the most relevant increase (about 69% and 124% at 14 and 21 days, respectively) compared to control, while the glutathione pool showed a prominent role mainly in roots, where the maximum increase in GSH content and a more pronounced increase of GSH/GSSG ratio were observed after 14 days of salt treatment [55]. This stress conditions also caused, especially in shoots, a general decrease in biomass production and a higher root/shoot ratio [55].

It is worth to note that the studies reported above investigated salinity-induced changes in antioxidant defence system by measurements of antioxidant enzyme activity and/or content in antioxidant molecules, as well as of gene expression. Interestingly, in the study of Capriotti et al. [56], a proteomic approach was used to evaluate the effect of salt stress (100 and 200 mM NaCl) on the salt- and drought-tolerant durum wheat cv. Duilio. As for the detoxification/defence system, significant changes in 9 proteins were found for the first stress level, in 10 proteins at the higher salinity level, and in 5 proteins when comparing salt-stress levels with each other, including some POX that result mainly upregulated for each salinity level. These results are also associated with a significant decrease of seedling weight (up to about −50%) and a seedling growth slower than in normal conditions with a relatively smaller reduction for the RWC at both salinity levels [56].

Some papers investigated the antioxidant defence system in durum wheat plants under combined application of salinity with other types of abiotic stress i.e., high light, high and low temperatures, metal toxicity (which often occur in the Mediterranean environment), showing that multiple environmental stresses could affect the stress response of plants. About this, changes in antioxidant system were studied in shoots of cv. Ofanto under the combined application of salt stress and high light, in comparison with low light [57]. In particular, SOD, APX, GR, and POX activities about doubled upon salinity in both light conditions, while CAT activity was halved by high light treatment, both alone or combined with salt stress. In addition, a strong decrease of both GSH (about −70%) and AsA (about −50%) levels as well as a significant increase of GSSG/GSH (about 2.2-fold) and DHA/AsA (about 2.4-fold) ratios were observed in leaves of plants under the two combined stresses compared with low light-salt stressed ones. The combined stress also decreased the water potential and stomatal conductance without reducing the photosynthetic efficiency of the plants [57]. Changes in CAT and GR activities, as well as in total AsA content, were examined also under combination of salt stress with normal (24/16 °C, day/night), high (40/30 °C), and low (5/−5 °C) temperatures in durum (cvs. Kızıltan-91, Kunduru 414-44 and Ç.1252) and bread wheat (cvs. Bezostaya-1, Seri-82, Kiraç-66) seedlings [51]. Under normal temperature, salt stress caused a significant increase of CAT activity in all genotypes, while high temperatures and salt stress combination induced a loss of CAT activity at higher levels in durum than bread wheat genotypes. Conversely, GR activity reached the highest value under high temperature-salt stress conditions [51]. Salinity, which increased total ascorbic acid level under normal temperatures, slightly increased it when applied in combination with both low and high temperatures. Moreover, the negative effects of salt stress on shoot and root elongation were forced by low and high temperature stress [51].

**Table 2:** Changes in antioxidant defence system in durum wheat under salt stress. Effects were estimated from graphs, figures and tables if not directly given in the text or supplements.

Plant Species	Growth Conditions	Stress Treatment/s	Tissue	Changes in Antioxidant Components	Ref.
**2 durum wheat genotypes** Om Rabia3 (salt-tolerant) Mahmoudi (salt-sensitive)	Glasshouse	50, 100, and 200 mM NaCl (imposed on 7-day-old seedlings) for 3 days	Shoot from 10-day-old seedlings	**SOD** activity: up to about 5- and 3-fold increase at 200 mM NaCl in Om Rabia3 and Mahmoudi, respectively **POX** activity: up to about 2- and 1.6-fold increase at 200 mM NaCl, in Om Rabia3 and Mahmoudi, respectively **CAT** activity: up to about 8- and 6-fold (increase at 200 mM NaCl in Om Rabia3 and Mahmoudi, respectively **APX** activity: up to about 2-fold increase at 100 mM NaCl in both genotypes **AsA** content: up to about −20% and −35% at 200 mM NaCl in Om Rabia3 and Mahmoudi, respectively Expression level of *CAT*, *MnSOD* and *APX* genes: from 2- to 4-fold increase	[52]
**2 durum wheat genotypes** Karim and Azizi	Hydroponic culture in growth chamber	100 mM NaCl for 11 days	Root and leaf from 21-day-old seedlings	**GSH** content: −82% and −64% in leaf and root of Karim, respectively, and −34% and −47% in leaf and root of Azizi, respectively **GSSG** content: no significant change in Karim and 51% decrease in root of Azizi	[53]
**2 durum wheat genotypes** Ofanto (drought tolerant) Adamello	Growth chamber	50 and 100 mM NaCl for 9 days	Root and shoot from 9-day-old seedlings	**APX**, **MDHAR**, **DHAR** and **GR** activities: general increasing and decreasing trends in shoots and roots, respectively **AsA**, **DHA**, **GSH**, and **GSSG** contents: variable, depending on genotype, tissue, and stress intensity	[54]
**1 durum wheat genotype** Ofanto (drought tolerant)	Hydroponic culture in growth chamber	20% (*v*/*v*) sea water for 14 and 21 days	Root and shoot from 14- and 21-day-old seedlings	**APX** and **GR** activities: variable depending on tissue and stress duration **AsA**, **DHA**, **GSH**, **GSSG** contents: variable depending on tissue and stress duration	[55]
**1 durum wheat genotype** Duilio	Hydroponic culture in growth chamber	100 and 200 mM NaCl (imposed on 5-day-old seedlings) for 10 days	Leaf from 15-day-old seedlings	**POXs** proteomic analysis: 1 isoform upregulated at both salinity levels, 1 isoform upregulated at 100 mM NaCl and 1 isoform downregulated at 100 mM NaCl	[56]
**1 durum wheat genotype** Ofanto (drought tolerant)	Hydroponic culture in phytotron	100 mM NaCl (imposed on the 10th day of hydroponic culture) for 10 days, the last 5 of which in combination with high light (900 μmol m^−2^ s^−1^ PAR)	Shoot from 20-day-old seedlings	**APX**, **GR** and **SOD** activities: about 2.2-fold increase under salt stress and low light **CAT** activity: about 1.7-fold increase under salt stress and low light **POX** activity: about 2.1-fold increase under salt stress and low light **AsA** content: 2.75-fold increase under salt stress and low light **GSH** and **GSSG** contents: 3.47-fold and 2.1-fold increase under salt stress and low light, respectively For combined effects of salinity and high light, see literature	[57]
**1 durum wheat genotype** AS 780 (salt-tolerant) **1 emmer wheat genotype** AS 847 (salt-sensitive)	Growth chamber	10 and 100 mM NaCl (imposed on 1 month-old seedlings for 2 weeks), supplemented with 3 mM MnSO_4_, for two weeks	Leaf	**APX** activity: 5-fold and +85% increase at 100 mM NaCl in durum wheat and emmer, respectively **GR** activity: +68% at 100 mM NaCl in both durum wheat and emmer **SOD** and **DHAR** activities: no significant change under salt stress For combined salt-manganese effects, see literature	[58]
**3 durum wheat genotypes** Kızıltan-91, Kunduru 414-44 and Ç.1252 **3 bread wheat genotypes** Bezostaya-1, Seri-82 and Kıraç-66	Growth chamber	0.7% NaCl (imposed on 6-day-old seedlings), in combination with low (5/−5 °C day/night) or high (40/30 °C) temperatures, for 6 days	Leaf from 12-day-old seedlings	**CAT** activity: about 2-fold increase in Kızıltan-91 and Kunduru 414-44 and about +30% in Ç.1252 under salt stress and normal temperature **GR** activity: slight effect under salt stress and normal temperature **AsA+DHA** content: about 2.4-fold increase in Kızıltan-91 and about +30% in Kunduru 414-44 and Ç.1252 under salt stress and normal temperature For combined effects of salinity and low or high temperatures, see literature	[51]
**2 durum wheat genotypes** Mohamed Ben Bachir (relatively drought-tolerant) and Hedba 3 (relatively drought-sensitive)	Growth chamber	10 g/L NaCl (imposed on 3-day-old seedlings), supplemented with 20 mM proline, for 10 days	Shoot from 13-day-old seedlings	**SOD** activity: 3- and 2-fold increase under salt stress in Hedba 3 and Mohamed Ben Bachir, respectively For combined salt-proline effects, see literature	[59]
**5 durum wheat genotypes** F7 recombinant inbred lines derived from a cross between durum wheat cv. Langdon and wild emmer wheat accession G18-16	Growth chamber	50 mM NaCl, supplemented with Na_2_SeO_4_ or Na_2_SeO_3_ at different concentrations (0.1, 1, 2, 4, 8, 10 μM), for 10 days	Root and shoot from 10-day-old seedlings	**SOD** activity: on average −12% under salt stress in shoots **CAT** activity: on average −9.5% and −9.7% under salt stress in roots and shoots, respectively **POX** activity: on average −5.5% and −4.8% under salt stress in roots and shoots, respectively For combined salt-selenium effects, see literature	[60]
**1 durum wheat genotype** Svevo	Hydroponic culture in growth chamber	200 mM NaCl (imposed on 10-day-old seedlings), combined with foliar treatment with chitosan (100 mg/L), for 7 days	Shoot from 17-day-old seedlings	**SOD** activity: +89% under salt stress **CAT** activity: +86% under salt stress **POX** activity: slight increase under salt stress For combined salt-chitosan effects, see literature	[61]

The effect of salt stress was investigated also in combination with MnSO_4_ stress by comparing the adaptive response of the salt tolerant durum wheat genotype (AS 780) to that of salt sensitive emmer wheat (AS 847) [58]. In particular, 2-week salt treatment increased APX and GR activities at a much higher extent in durum wheat genotype than emmer. Mn treatment alone induced a significant increase in SOD, APX, GR, and DHAR activities in both genotypes, with higher values of SOD and APX in durum wheat. Compared to Mn stress, the combined stress further increased SOD, APX, and GR activities in durum wheat, but inhibited SOD, APX, and DHAR in emmer. Combined stress also affected negatively photosynthesis, plant growth, and RWC, and increased ROS production and MDA content at a higher extent in emmer than in durum wheat, thus indicating the superior adaptability to salt-Mn combined stress of durum wheat compared to emmer [58].

Interestingly, some reports investigated the capability of exogenously applied compounds in improving durum wheat adaptability of salt stress, by mitigating the adverse effects of salinity. Sensitivity to proline alleviation of salt stress was investigated in the Algerian durum wheat varieties Hedba 3 (relatively drought-sensitive) and Mohamed Ben Bachir (relatively drought-tolerant) [59]. A three-fold increase of SOD activity under salt stress and a more than four-fold increment under salt stress-proline combination were observed in Hedba 3, while no significant changes under salt and proline or both treatments were measured in Mohamed Ben Bachir [59]. Moreover, salt stress induced in Mohamed Ben Bachir a significant decrease of leaf RWC and an increase of MDA content as well as a differentially organ growth inhibition (as roots were more sensitive than shoots) in both cvs. Nevertheless, when proline was present during salt stress, both RWC and MDA remained at the control level and growth was partially restored in both genotypes. The possible role of selenium in mitigating salt-induced oxidative stress was also investigated in roots and shoots obtained from five F7 durum wheat recombinant inbred lines [60]. Addition of low concentrations (0.1–4 μM) of sodium selenate or sodium selenite to the NaCl solution increased CAT, SOD and POX activities in roots and shoots compared to salt stress alone, while a decrease was observed at higher selenium salt concentrations (8–10 μM). Interestingly, at low concentrations, selenium also mitigated the adverse effects of salt stress on seed germination, biomass accumulation, decreased the concentrations of MDA and had positive effects on chlorophyll fluorescence indices, thus suggesting that selenium can improve the adaptability of durum wheat to salt stress [60]. Quitadamo et al. [61] evaluated the protective role of chitosan against the oxidative stress induced by salinity in shoots of durum wheat cv. Svevo. Salt stress induced a 5-, 2-, and 4-fold increase of O_2_·^−^ generation rate, H_2_O_2_ and MDA content, respectively, highly correlated with the reduction of dried biomass, as well as an increase of SOD, CAT, and POX activities of about 90%, 85%, and 15%, respectively. Interestingly, the treatment of salt-stressed seedlings with exogenous chitosan significantly promoted seedling growth and decreased ROS production, as well as MDA content [61].

In the whole, similarly to drought, a reverse behaviour of the antioxidant system compared with biomass and physiological traits was generally exhibited. Moreover, these studies highlighted a complex response of the antioxidant system showing an overall increase under salt stress, which may be attributed differently to the different components of the antioxidant network depending on the genotype, the applied experimental conditions (i.e., duration and intensity of stress, time of stress imposition, stress combination) and the investigated tissue/organ.

## 3. Conclusions

Considering the global climate change scenario, the main obstacle to food availability is sustained loss of crops due to abiotic stresses, particularly drought and salinity. In the past decades, a great progress has been made in understanding how drought and salinity stresses may affect plant growth and yield, and how the plant responds/adapts to these stresses. In particular, a relevant interest has been addressed to research on the antioxidant defence system in plants, because the tolerance of some genotypes to environmental stresses has been associated with higher activities of antioxidant enzymes [36,40,42].

Overall, results of the studies discussed in this review clearly show that the enhancement of the antioxidant defence system plays, also in durum wheat, a pivotal role in plant adaptation to hyperosmotic stress and represents an important strategy in the drought and salt tolerance mechanisms for this crop. In fact, although this plant species has been less investigated than other cereals, relevant changes in levels of key antioxidant enzymes and molecules have been reported in durum wheat plants in response to salt stress and water deficiency.

**Figure 1:**
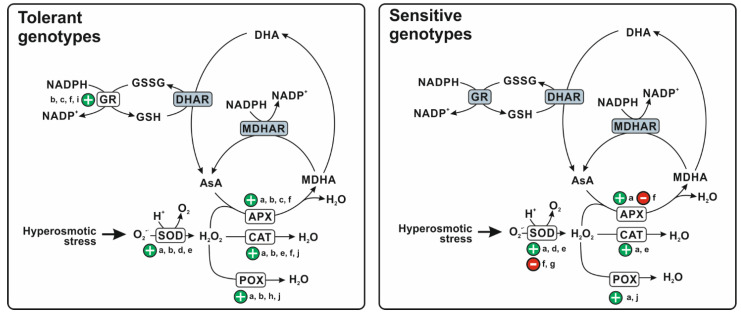
Changes in the antioxidant defence system in shoots/leaves of tolerant and sensitive durum wheat genotypes. SOD, superoxide dismutase; APX, ascorbate peroxidase; CAT, catalase; POX, peroxidases, MDHAR, monodehydroascorbate reductase; DHAR, dehydroascorbate reductase; GR, glutathione reductase; AsA, reduced ascorbate; MDHA, monodehydroascorbate; DHA, dehydroascorbate, GSH, reduced glutathione; GSSG, oxidized glutathione. + and – signs refer to upregulation and downregulation of the enzymes, respectively. For the enzymes highlighted in gray, no sufficient data are available. The stoichiometry of the reactions is not always respected. (a) Feki et al. [52]; (b) Woodrow et al. [57]; (c) Sheng et al. [58]; (d) Ami et al. [59]; (e) Ayadi et al. [44]; (f) Huseynova [46]; (g) Huseynova et al. [47]; (h) Capriotti et al. [56]; (i) Meneguzzo et al. [54]; (j) Huseynova et al. [48].

These changes are highly influenced by genotype. Indeed, durum wheat plants showing different drought/salt tolerance respond differently to hyperosmotic stress in terms of activities of antioxidant enzymes and/or levels of antioxidant molecules. Based on literature data reported in this review, a possible pattern of changes of the different antioxidant enzymes induced by hyperosmotic stress in shoot/leaves of both sensitive and tolerant durum wheat genotypes is suggested in Figure 1.

Most of literature studies agree in showing the stress-induced activation in the tolerant genotypes of SOD enzyme, responsible of superoxide anion dismutation, as well as of the H_2_O_2_-scavenging enzymes APX, CAT, and POX. On the other hand, only few papers report the activation of the same enzymes in the sensitive genotypes, although with a much lower magnitude than the tolerant ones, or even their downregulation. Moreover, GR, involved in H_2_O_2_ removing by maintaining favourable levels of GSH, resulted to be upregulated under hyperosmotic stress in the tolerant plants, while the effect of drought and salinity on this enzyme has not been sufficiently investigated in the sensitive genotypes. In the light of this different behaviour, drought- and salt-tolerant durum wheat cultivars may maintain a higher efficiency of antioxidant protective system under hyperosmotic stress as compared to the sensitive genotypes, and consequently display a higher capability to alleviate ROS-induced oxidative damage. This may allow the tolerant genotypes to preserve the structural integrity of cell components and, in particular, of photosynthetic apparatus, thus maintaining an efficient photosynthetic activity and growth rate. It should be outlined that changes in antioxidant behaviour of durum wheat under hyperosmotic stress also depend on the severity and duration of the stress, on the stage of development at which the stress is imposed, as well as on the different plant tissues/organs investigated. Although very little information is available about these aspects, relevant changes in antioxidant response may be suggested to occur in the flowering and ripeness stages, when plants are more exposed and sensitive to hyperosmotic stress.

Moreover, different patterns in antioxidative molecules and enzymes may be also observed in shoots, leaves, and roots, thus suggesting that different kinds of reaction to the stress occur in different tissues/organs of durum wheat plants under hyperosmotic stress. Distinct variations of activities of enzymes performing the neutralization of the same ROS, as well as of isoforms with different subcellular/tissue localization and/or expressed in different developmental stages, have been also described under hyperosmotic stress. This heterogeneity of forms can represent a very valuable feature able to extend plant adaptability against constantly changing environmental conditions. Moreover, some studies showed important differences in hyperosmotic stress response of durum wheat in the case of combination of hyperosmotic stress with other environmental stresses, thus suggesting that further research is required to better understand the behaviour under multiple environmental stress factors, to which this crop is frequently subjected in the Mediterranean environments.

In the whole, these studies highlighted the complexity of the response of the antioxidant system that acts as a coordinated network of synergically working enzymatic and non-enzymatic components, where deficiencies in one component may affect the efficiency of the others in maintaining the equilibrium between ROS production and detoxification at cellular level.

Further investigations are needed for a comprehensive understanding of relationships of antioxidant status with physiological and yield traits involved in durum wheat response to water deficit and soil salinity. This multidisciplinary approach is necessary for improvement of durum wheat adaptation and productivity under drought/salt stress through the development of new tolerant and high-yielding genotypes for sustainable production under the upcoming environmental limitations.
